# Trade-offs between cold protection and air pollution–induced mortality of China's heating policy

**DOI:** 10.1093/pnasnexus/pgad387

**Published:** 2023-11-11

**Authors:** Haofan Zhang, Pan He, Linxin Liu, Hui Dai, Bin Zhao, Yi Zeng, Jun Bi, Miaomiao Liu, John S Ji

**Affiliations:** State Key Laboratory of Pollution Control and Resource Reuse, School of the Environment, Nanjing University, Nanjing 210023, China; School of Earth and Environmental Sciences, Cardiff University, Cardiff CF24 4AT, UK; School of Earth and Environmental Sciences, Cardiff University, Cardiff CF24 4AT, UK; Vanke School of Public Health, Tsinghua University, Beijing 100084, China; Department of Building Science, School of Architecture, Tsinghua University, Beijing 10084, China; Department of Building Science, School of Architecture, Tsinghua University, Beijing 10084, China; Center for Healthy Aging and Development Studies, Raissun Institute for Advanced Studies, National School of Development, Peking University, Beijing 100871, China; Center for the Study of Aging and Human Development and Geriatrics Division, Medical School of Duke University, Durham, NC 27708, USA; State Key Laboratory of Pollution Control and Resource Reuse, School of the Environment, Nanjing University, Nanjing 210023, China; State Key Laboratory of Pollution Control and Resource Reuse, School of the Environment, Nanjing University, Nanjing 210023, China; Vanke School of Public Health, Tsinghua University, Beijing 100084, China

**Keywords:** winter heating, China, health risk assessment, air pollution, protection against low temperatures, mortality, older adults

## Abstract

The winter heating policy in northern China was designed to safeguard households from the harsh subfreezing temperatures. However, it has inadvertently resulted in seasonal spikes in air pollution levels because of the reliance on coal as an energy source. While the loss of life years attributable to mortality from air pollution caused by winter heating has been estimated, the beneficial effect of protection from cold temperatures has not been assessed, primarily due to a lack of individual-level data linking these variables. Our study aims to address this research gap. We provide individual-level empirical evidence that quantifies the impact of protection from cold temperatures and air pollution on mortality, studying 5,334 older adults living around the Huai River during the period between 2000 and 2018. Our adjusted Cox-proportional hazard models show that winter heating was associated with a 22% lower mortality rate (95% CI: 16–28%). Individuals residing in areas without access to winter heating are subjected to heightened mortality risks during periods of cold temperatures. The protective effect is offset by a 27.8% rise attributed to elevated PM_2.5_ levels. Our results imply that the equilibrium between the effects of these two factors is achieved when PM_2.5_ concentration exceeds 24.3 µg/m^3^ (95% CI: 18.4–30.2). Our research suggests that while the existing winter heating policy significantly mitigates winter mortality by lessening the detrimental effects of cold temperatures, future air pollution reduction could provide further health benefits.

**Significance Statement**

China’s winter heating is a cause of air pollution emissions. However, there is an unknown trade-off between air pollution and protection against cold temperatures on health. Using individual-level data in a prospective cohort, we evaluated the long-term effects of winter heating in China. Our findings revealed a significant association between winter heating and longevity. At the same time, we also established that the protective benefits of winter heating are compromised when heating-related PM_2.5_ concentrations hit 24.3 µg/m^3^.

## Introduction

Winter heating–induced air pollution in northern China has been recognized as a significant contributor to premature mortality. For 3–4 months during the winter, millions of individuals in this region endure subzero temperatures (1). In response, China implemented a winter heating policy to safeguard people who experience subzero temperatures during winter (2). However, the resultant heating predominantly relies on coal power, with coal-fired heating areas accounting for about 83% of total heated areas (3). During winter, large centralized coal-fired boilers burn 200 million standard coal to provide indoor heating in urban areas of northern China. Rural residents of northern China burn the same amount of raw coal for dispersed small residential heating stoves and boilers (4). Consequently, the winter months in northern China witness a five-fold deterioration in air quality compared with the nonheating seasons (5).

The impact of heating-related air pollution on life expectancy in northern China is substantial. Estimates suggest that the residents of northern China lost 2.5 billion life years of life expectancy because of heating-related air pollution (6). Further, an econometrics study using a quasi-experimental (geographic regression discontinuity) design attributed 3.7 billion life years of life expectancy losses in northern China to winter heating–related air pollution (7), while another analysis associated a 14% increase in weekly mortality rate with the onset of winter heating (8).

Notwithstanding these findings, previous studies have overlooked the potential protective role of winter heating against the health risks posed by low temperatures. The negative health impact of air pollution has been well-documented; however, there has been a lack of research into the possible reduction in cold-related mortality conferred by winter heating. A comprehensive understanding of these trade-offs requires an analysis of individual-level data in an epidemiologic cohort study. This approach would provide real-world evidence to inform better policy decisions surrounding winter heating in China.

Our study aims to fill this evidence gap by examining the winter heating status–mortality, air pollution–mortality, and cold temperature–mortality relationship in Chinese older and oldest old adults based on a nationally representative longitudinal cohort study. First, the longitudinal study design and detailed individual information allow us to estimate long-term heating and air pollution exposure and reduce confoundings. Second, we performed a mediation analysis to investigate the effect of winter heating on mortality mediated by heating-related air pollution. Third, we focused on participants in five provinces around the *Huai River Line* (winter heating boundary) where the temperature difference is slight and physiological adaptations of residents may be comparable. Fourth, we compared long-term and acute cold temperature mortality risk between participants with and without winter heating to explore how heating affects health by modifying the risk of temperature-related mortality.

## Results

We analyzed a total of 7,199 older adults in 5 provinces around the Huai River during the period between 2000 and 2018. For the long-term effect analysis, participants with missing information on temperature (*n* = 283), PM_2.5_ (*n* = 259), greenness (*n* = 220), and other covariates (*n* = 563), as well as those who died or were lost to follow-up before the 2018 survey (*n* = 540), were excluded. The final analysis included 5,334 participants with a mean age of 85.7, resulting in 34,608 person-years of follow-up. Of these, 1,813 (34.0%) lived in areas with winter heating. The mean winter temperature for those living in these areas was 2.57°C with a temperature variability of 4.40°C, which was slightly lower than for those living in nonheating areas. Participants living in winter heating areas were older, more likely to have formal education, be financially dependent, and have higher exposure to ambient PM_2.5_ at baseline. The mean (standard deviation) exposure levels of PM_2.5_ for participants in winter heating and nonheating areas were 91.90 (16.36) μg/m^3^ and 79.22 (13.93) μg/m^3^, respectively (Table S1). For the daily temperature and mortality, we recorded 6,667 deaths with available temperature, and 2,051 (30.8%) occurred in winter. Of these participants, 2,070 (31.0%) lived in areas with winter heating. The maximum temperatures on the day of death for participants living in heating and nonheating areas were 18.1 and 19.4°C, respectively (Table S2).

In the Cox-proportional hazard model–adjusted age, gender, ethnicity, urban/rural residence, education, marital status, financial support, smoking status, alcohol consumption, and physical activity, the protective effect of heating was not statistically significant, with the mortality hazard ratio (HR) for winter heating being 0.95 (95% CI: 0.89–1.02; Table 1, Model 1). When we further adjusted the individual seasonal mean temperature and seasonal temperature variability, the HR for winter heating became 0.78 (95% CI: 0.72–0.84; Table 1, Model 4).

**Table 1.** Associations of heating in winter and mortality.

	Model 1	Model 2	Model 3	Model 4	Model 5	Model 6
Heating (vs. nonheating)	0.95	0.801^a^	0.826^a^	0.778^a^	0.743^a^	0.707^a^
	(0.035)	(0.038)	(0.043)	(0.042)	(0.044)	(0.043)
PM_2.5_ (10 µg/m^3^)	**—**	1.160^a^	**—**	**—**	1.131^a^	1.121^a^
		(0.012)			0.012	(0.012)
Annual average temperature (1°C)	**—**	**—**	1.067^a^	**—**	1.065^a^	**—**
			(0.018)		0.018	
Annual temperature variability (1°C)	**—**	**—**	1.465^a^	**—**	1.379^a^	**—**
			(0.031)		0.032	
Summer average temperature (1°C)	**—**	**—**	**—**	1.270^a^	**—**	1.247^a^
				(0.020)		(0.020)
Summer temperature variability (1°C)	**—**	**—**	**—**	1.119^a^	**—**	1.146^a^
				(0.033)		(0.330)
Winter average temperature (1°C)	**—**	**—**	**—**	0.863^a^	**—**	0.880^a^
				(0.011)		(0.011)
Winter temperature variability (1°C)	**—**	**—**	**—**	1.185^a^	**—**	1.162^a^
				(0.019)		(0.019)

All models adjusted age, gender, ethnicity, urban/rural residence, education, marital status, financial support, smoking status, alcohol consumption, physical activity, and residential greenness. Model 1 included heating status. Model 2 further adjusted residential PM_2.5_ based on Model 1. Model 3 further adjusted the annual average temperature and annual temperature variability based on Model 1. Model 4 further adjusted summer average temperature, summer temperature variability, winter average temperature, and winter temperature variability based on Model 1. Model 5 further adjusted residential PM_2.5_, annual average temperature, and annual temperature variability based on Model 1. Model 6 further adjusted residential PM_2.5_, summer average temperature, summer temperature variability, winter average temperature, and winter temperature variability based on Model 1 (^a^*P* < 0.001).

**Fig. 1.**
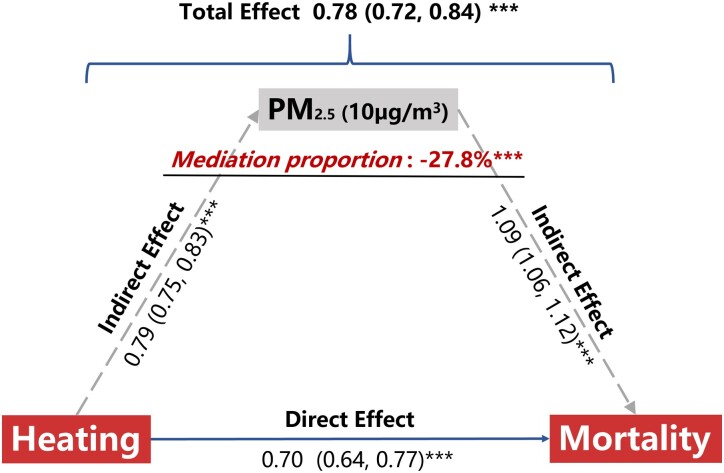
Winter heating's mediating effects on all-cause mortality through PM_2.5_. The total effect of winter heating on all-cause mortality was 0.78. The direct effect of winter heating on all-cause mortality was 0.70. Mediation proportion means the dimensionless proportion of the effect of winter heating on mortality mediated through PM_2.5_. A 10 µg/m^3^ increase in PM_2.5_ was associated with a 9% higher risk of all-cause mortality. Participants in the heating areas were exposed to 7.9 µg/m^3^ higher PM_2.5_ levels than those in the nonheating areas.

Our mediation provided additional insight: the total protective effect, including both direct and indirect effects of winter heating on mortality, was estimated at 0.78 (95% CI: 0.72–0.84; Figure 1). Our results revealed that winter heating was associated with increased levels of PM_2.5_, and the mediation analysis showed that the indirect effect of winter heating on survival time through the mediator PM_2.5_ accounted for −27.8% (*P* < 0.001) of the total effect, because of the opposite direct and indirect effects. The direct protective effect of winter heating on all-cause mortality was estimated to be 0.70 (95% CI: 0.64–0.77). Our analysis showed that air pollution attenuated only a part of the protective effects of heating, which was the most significant finding from this study.

**Fig. 2.**
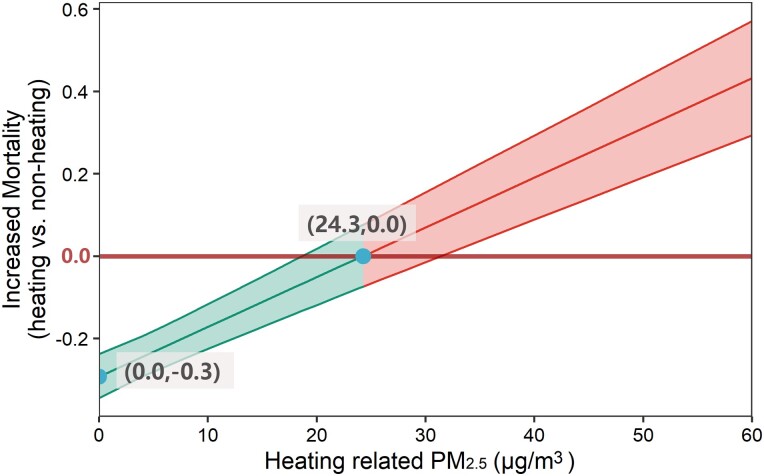
Trade-offs between the direct effect of winter heating on mortality and the increased risk of heating-related PM_2.5_. When heating-related PM_2.5_ equals 0, the increased mortality caused by heating-related PM_2.5_ (heating areas vs. nonheating areas) shows the direct effect of heating on mortality, which is heating reduced the risk of mortality for participants in heating areas by 30% compared with participants in nonheating areas. Increased mortality caused by heating-related PM_2.5_ is <0, which means that winter heating has a protective effect considering both the directed effect of heating and the indirect effect of the increased PM_2.5_ caused by winter heating. When heating-related PM_2.5_ equals to 24.3 µg/m^3^, the protective effect of heating is completely offset by the increased PM_2.5_.

**Fig. 3.**
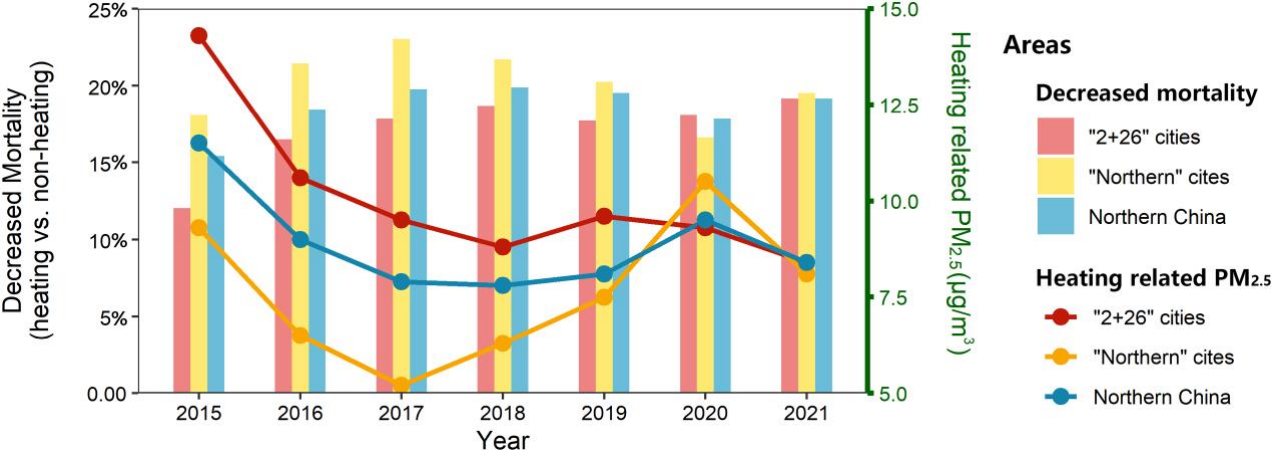
Temporal variations of the sum of the direct effect of winter heating and the increased risk of heating-related PM_2.5_ on mortality. The right axis presents existing data detailing the annual average contribution of winter heating to PM_2.5_ levels each year. In contrast, the left axis illustrates how winter heating has decreased mortality rates in heated areas. The heating-related PM_2.5_ is taken from Song et al. (i) The term “2 + 26” cities refers to those that have implemented clean heating measures. (ii) “Northern” cities denote the cities in northern China, excluding the aforementioned “2 + 26” cities.

Using Cox and mediation models, we explored the net effect resulting from the direct protective impact of winter heating on mortality and the heightened risk linked to heating-related air pollution. Our analysis revealed a balance point: the protective advantage of heating was offset by the risk posed by heating-related PM_2.5_ when PM_2.5_ concentrations rose to 24.3 µg/m^3^ (95% CI: 18.4–30.2), as depicted in Figure 2. Furthermore, we incorporated a recent quasi-natural experimental study that utilized a machine learning–based weather normalization method to estimate the causal impacts of winter heating on air quality (9). Our results suggest that, from a population health standpoint, the benefits of China’s winter heating policy outweighed the harms (see Figure 3). When we extrapolated the relationship among winter heating, PM_2.5_ levels, and mortality, we deduced that the winter heating policy was associated with a decrease in mortality rates in northern China, from 15.4% in 2015 to 19.6% in 2021.

Participants without winter heating faced a higher mortality risk in both long-term and short-term cold temperatures compared with those with winter heating (Table S3 and Figure S1). For the long-term temperature exposure model, the HR for each 1°C increase in winter temperature variability was higher for participants without winter heating than for participants with winter heating (Table S3, 1.21 vs. 1.08), but the HR for each 1°C increase in summer mean temperature, winter mean temperature, summer temperature variability, and each 10 µg/m^3^ increase was similar (Table S3). For the daily temperature exposure model, the relative risk (RR) of mortality at 3°C (5^th^ percentile) compared with 23°C (65^th^ percentile) for participants in winter heating and nonheating areas was 1.14 and 1.48, respectively (Figure S1). However, we found no difference in heat mortality risks between participants with or without winter heating. The RR of mortality at 34°C (95^th^ percentile) compared with 23°C (65^th^ percentile) for participants in winter heating areas and nonheating areas was 1.07 for both areas.

Our sensitivity analysis showed when adjusting residential PM_2.5_ and seasonal temperature averages and variability, winter heating reduced the risk of mortality by 42% for urban residents and 25% for rural areas (Figure S3). When we further characterized air pollution exposure by using indoor PM_2.5_, the average of indoor and outdoor PM_2.5_, and a weighted average of indoor and outdoor PM_2.5_ and adjusted it in models, we found that winter heating reduced the risk of mortality by 22, 23, and 21%, respectively (Table S4).

## Discussion

This study provides a comprehensive understanding of the health risks associated with winter heating. We observed a substantial reduction of 22.2% in all-cause mortality among individuals who had winter heating despite their higher exposure to PM_2.5_ in heating areas. Both rural and urban residents gained from winter heating once air pollution adjustments were made. However, the benefits for rural residents were less pronounced than those for urban residents. This might be attributed to the indoor air pollution generated by self-sufficient heating in rural regions. Indeed, emissions from residences accounted for 71% of the indoor PM_2.5_ concentration (10), particularly in rural areas, where solid fuels accounted for 82% of heating energy usage (11). While other studies do not consider air pollution simultaneously, several studies have investigated the health benefits of winter heating (12–14). Cold weather has a direct effect on the incidence of hypothermia, heart attack, stroke, respiratory disease, and flu (15, 16). The possible biological mechanism may be caused by the human body’s stress response in thermal regulation (17). Exposure to cold temperatures will elicit acute physiological responses, including cutaneous vasoconstriction, shivering thermogenesis, and heart rate change to defend the core temperature. A prolonged stress response can damage the body’s functions (18). Heating provides people with a comfortable indoor thermal environment and avoids exposure to cold, lightening the physical stress response and decreasing mortality risk (19).

To validate the assumption that winter heating reduces mortality by alleviating cold-related risks, we performed a stratified analysis to examine the relationship between temperature and mortality. Our findings indicated that participants with winter heating had lower mortality risks associated with increased winter temperature variability and extremely cold temperatures compared with those without heating. Temperature variabilities serve as long-term indicators of weather conditions, reflecting fluctuations in temperature levels to which individuals have become accustomed over time (20). Higher winter temperature variability, summer temperature variability, summer mean temperature, and lower winter mean temperature were significantly related to shorter survival time (21, 22). Our analysis revealed both similarities and differences in the relationships between seasonal temperature indicators and mortality among participants with and without winter heating. These findings suggest that winter heating can enhance adaptability to temperature changes during the winter season. Cold daily temperatures are associated with increased mortality risk (23, 24). Consistent with this, our analysis also demonstrated significant RRs for cold temperatures in nonheating areas. However, in heating areas, the effects of cold temperatures were minimal, indicating that winter heating could effectively reduce mortality risks associated with extremely low temperatures.

We quantified the contribution of heating-related air pollution to the overall health effects of winter heating. The protective effect of winter heating on all-cause mortality increased when accounting for PM_2.5_ levels, suggesting that the air pollution resulting from winter heating partially overshadowed the health benefits associated with heating. Our mediation model offered quantitative insights, revealing that increased air pollution accounted for 27.8% of the diminished longevity benefits of heating. Compared with previous regression discontinuity studies that focused on the relationship between air pollution and health outcomes, our study provides a comprehensive perspective on the interrelationship between winter heating, health, cold temperatures, and related air pollution.

Based on our findings, if the level of PM_2.5_ attributable to winter heating remains below 24.3 μg/m^3^, considering both the protective effect of heating and the increased PM_2.5_, the policy will lead to a decrease in mortality. In 2017, China introduced the “Clean Heating Plan in Northern Region,” which officially made clean heating a national strategy. This plan involves the use of high-efficiency clean energy as a substitute for low-efficiency coal boilers. It also includes the development of a heat supply network and the construction of energy-saving buildings, aiming to achieve heating with low emissions and low pollution (4). Clean heating policies have improved the annual (9) and winter air quality (25) in northern China and bring billions of economic benefits (26), especially in rural areas (27). The findings from this study strongly indicate that China’s winter heating policy has yielded significant health benefits. Our comprehensive assessment of the health risks associated with winter heating can inform a thorough evaluation of the clean heating project and contribute to a more informed assessment of the efficacy and potential impact of the clean heating initiative.

Our study has certain limitations that should be acknowledged. First, we were unable to directly calculate the PM_2.5_ concentration specifically attributable to winter heating using our data set. Additionally, our data had a limited sample size extracted from real-world evidence–presented challenges with a symmetrical alignment on either side of the heating line, which restricted our analysis from studying and implementing other analytical methods such as regression discontinuity design. However, we drew upon the findings of a recent study, which provides causal evidence on the impacts of winter heating on air quality, to illustrate the overall health effects of winter heating. We demonstrated the population-level benefits of winter heating for health. Second, we had only ambient temperature data and no indoor temperature data, making it impossible to directly prove that winter heating protects health by providing a comfortable indoor thermal environment. We indirectly demonstrated this by confirming the modification effect of heating on the relationship between outdoor temperature and mortality. Third, we did not have a sufficient number of participants to explore the modification of age group, gender, and marital status on the protective effect of heating in death, considering the limitations of statistical power.

## Conclusion

Our findings suggest that China’s current winter heating policy is crucial in curtailing winter mortality by counteracting the harmful effects of cold temperatures despite the concurrent rise in air pollution. Ignoring the protective influences of winter heating against cold temperatures restricts our comprehension of the overall health impact. Future research ought to contrast the protective role of heating against low temperatures and the health risks posed by air pollution across varying populations to spotlight disparities. If cleaner energy sources or enhanced energy efficiency are incorporated into the winter heating policy, there could be potential for augmented temperature protection benefits, particularly for China’s elderly population.

## Materials and methods

### Study population and cohort design

**Fig. 4.**
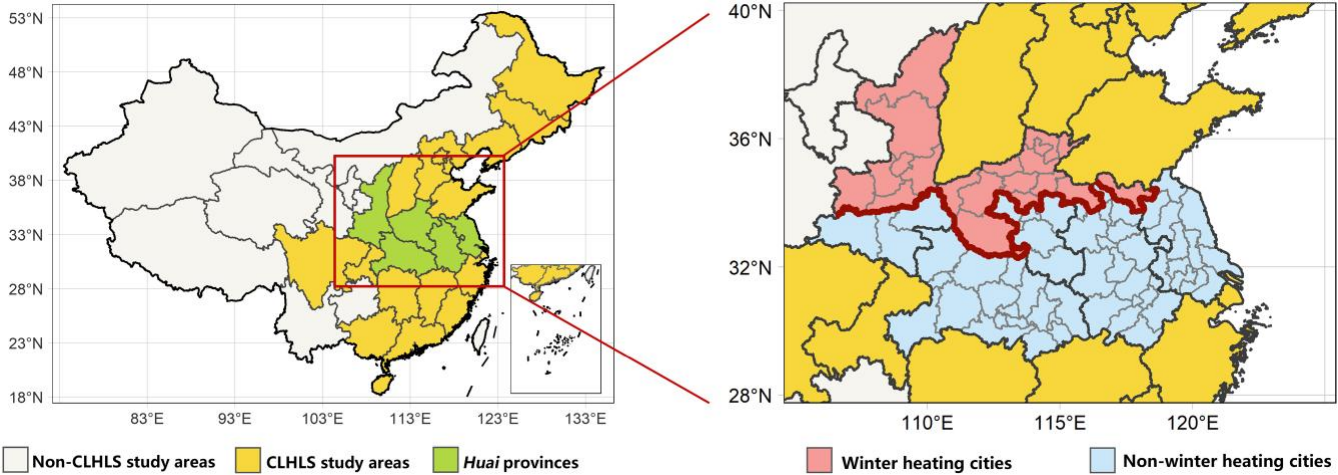
Maps of the study areas. Huai Provinces: Five provinces between heating and nonheating areas (Jiangsu, Anhui, Henan, Hubei, and Shaanxi).

The Chinese Longitudinal Healthy Longevity Survey (CLHLS) is a nationwide prospective open cohort designed to explore healthy longevity determinants. With a multistage and stratified sampling design, older adults from 22 of 31 provinces in China were individually interviewed about demographic characteristics, socioeconomic status (SES), lifestyle, physical and mental health, and mortality every 2 years since 1998. The CLHLS was approved by the research ethics committees of Duke University and Peking University (IRB00001052-13074). All participants gave informed consent. Details of the CLHLS have been described elsewhere (28). We used the 2000–2018 survey data and focused on participants living in five provinces between heating and nonheating areas (Jiangsu, Anhui, Henan, Hubei, and Shaanxi; Figure 4), referred to as “Huai Provinces” in this paper. Restricting the study area aims to reduce the differences in physiological adaptability caused by differences in long-term exposures.

### Exposure assessment

We obtained city-level heating conditions from the China Urban Construction Statistical Yearbook and the websites of local governments. The primary analysis assumed that rural (residing in the county) residents were also available for winter heating in a heated city, considering the self-sufficient heating.

Daily monitoring data from 2,456 meteorological stations across China during the period between 1999 and 2019 were obtained from the China Meteorological Administration. We assigned the nearest meteorological station data to each participant based on their residence. For long-term temperature exposure, we used the daily maximum temperature to calculate annual and seasonal (summertime: June–August, wintertime: December–February) mean temperature and temperature variability (standard deviation of the temperatures) for each participant in each year. The annual and seasonal mean temperature and temperature variability have been demonstrated to play a significant role in raising the mortality risk. For acute temperature exposure, we used lagged daily maximum temperature before the death of participants.

The annual mean concentration of satellite-based PM_2.5_ at 0.1° resolution over the study period was obtained via remote sensing methods consistent with prior studies (29). These methods have been demonstrated to have a minor random error but are generally considered reliable (30, 31). We matched the grid PM_2.5_ concentration to each participant's residential location. A previous study showed that 3-year annual PM_2.5_ before the event had the strongest association with mortality of the fifth wave of the CLHLS (32). Here, we chose the exposure windows as 1 year, considering the availability of historical PM_2.5_ data and the study population. To evaluate the overall health impact of the winter heating policy, we used the latest quasi-natural experimental assessments of heating-related air pollution from Song et al (9). They constructed an artificial city without winter heating that is comparable with a city with winter heating by employing an augmented synthetic control method. They also decoupled the effects of meteorology from observed air pollutant concentrations, applying a random forest–based weather normalization approach. This improved observation-based causal inference approach provides causal evidence on the impacts of winter heating on air quality.

We also estimated the individual’s residential greenness using the Normalized Difference Vegetation Index (NDVI), a satellite-image-based vegetation index. It ranges from −1.0 to 1.0, with larger positive values indicating higher vegetative density levels. We obtained NDVI measurements from the Moderate-Resolution Imaging Spectro-Radiometer (MODIS) in the National Aeronautics and Space Administration’s Terra Satellite and calculated the greenness around participants' residential addresses. We removed participants with negative NDVI values, which refer to blue space or water, considering that water surfaces may have independent beneficial effects on health. Consistent with our previous research, we used NDVI values in the 500 m radius around the residential address at the time closest to an event in the analyses (33).

### Statistical analysis

First, we used Cox-proportional hazard models to assess the effect of heating in winter on mortality. We further compared the mortality risks of long-term temperatures for participants with and without heating. Using Cox-proportional hazard models, we modeled long-term temperature exposure as a time-varying continuous variable with the HR and 95% CI estimated per 1°C increase in exposure. We simultaneously entered these four temperature exposure variables (summer mean temperature, winter mean temperature, summer temperature variability, and winter temperature variability) into the models to separate their independent associations with mortality (22, 34). It means that all other exposure indices were held constant when we looked at the effect of one exposure index. Also, the auto-correlation between these indices would not be an issue. The independent associations with mortality for these four variables were referred to as the overall effect for each exposure index.

The adjusted model included age, gender, ethnicity, urban/rural residence, education, marital status, financial support, smoking status, alcohol consumption, and physical activity. We calculated age according to interview dates and verified birth dates (1-year strata). Ethnicity was divided into two categories: Han Chinese and ethnic minority. The residence was dichotomized into urban, town, and county areas based on governmental administrative categories. We dichotomized education status into formal education (≥1 schooling year) and no formal education (<1 schooling year) because education attainment was not ubiquitous during the historical era of our study participants' adolescence. We generated a binary variable to assess marital status: married and living with a spouse or unmarried at the interview (separated, divorced, widowed, or never married). We divided the participants into financial independence (self-supported work and retirement wage) and financial dependence (financially relying on other family members). Smoking status was categorized into never smokers (nonsmoked in the past and at the interview), former smokers (smoked in the past but nonsmoked at the interview), and current smokers (smoked at the interview). We used a similar method to categorize alcohol consumption and physical activity.

Based on the acknowledged health risk of winter heating–related PM_2.5_, we designed a “winter heating—PM_2.5_—all-cause mortality” pathway hypothesis and performed a regression-based causal mediation analysis (35, 36) to evaluate the mediating effect of winter heating on all-cause mortality through PM_2.5_ and investigate the direct and indirect effects. We hypothesized no unmeasured exposure-outcome confounders, mediator-outcome confounders, or exposure-mediator confounders in the mediation analysis.

In addition, we used a time-stratified case-crossover study design with distributed nonlinear modeling to estimate the mortality risk for the daily temperature exposure (37). Case-crossover design can eliminate confounding bias from individual differences such as age, sex, SES, and health status. We selected control time windows as the same day of the week from the same calendar month of death in the same year. We used the daily maximum temperature for the day death occurred and control time windows for each individual. We then fitted the time-stratified case-crossover design with a Cox-proportional hazard regression model. We used distributed lag nonlinear models (DLNMs) to build the time structure of associations between temperature and mortality, considering the different lags of the temperature effect. The non-linear association between temperature and mortality was modeled by using a B-spline with 3 degrees of freedom, and knots were placed at the quartiles (25^th^, 50^th^, and 75^th^) of the temperature distribution. We presented results for the nonlinear association between temperature and risk of mortality and summary measures comparing two points on these curves. For heat, we considered the risk of mortality at the 95^th^ percentile of temperature compared with the risk at the 50^th^ percentile. For cold, we presented mortality risk estimates at the 5^th^ percentile of temperature compared with the 50^th^.

We conducted all analyses with R software (version 4.0.3) using the packages of survival, dlnm, and mvmeta, regmedint.

### Sensitivity analysis

We conducted several sensitivity analyses to validate the robustness of our primary findings. These included (i) limiting winter heating access to urban residences, (ii) substituting air pollution exposure representation with city-level indoor PM_2.5_ concentrations, and (iii) broadening our scope to encompass a nationwide population. The indoor PM_2.5_ concentration at the city level was procured from a recent study in which a Bayesian Neural Network (BNN) model was employed to predict indoor PM_2.5_ levels across the country in 2019. Our prediction relied on data from a wide range of sources, including comprehensive nationwide sensor-monitoring records in China. We trained our BNN model using real-time indoor PM_2.5_ monitoring data collected from 18,484 anonymized households located in 36 Chinese cities between November 2016 and July 2018. This model took into account multiple factors such as outdoor PM_2.5_ concentration, air temperature, relative humidity, wind speed, population, and Gross Domestic Product. The BNN model demonstrated high accuracy, with a 10-fold cross-validation *R*^2^ of 0.70, a mean absolute error of 9.45 μg/m^3^, a root-mean-square error of 13.3 μg/m^3^, and a 95% prediction interval coverage of 85%.

## Supplementary Material

pgad387_Supplementary_Data

## Data Availability

The data that support the findings of this study are available on request from authors. CLHLS is publicly available from data repository, some file access isrestricted.
