# Supplementary material for: Trade-offs between cold protection and air pollution–induced mortality of China's heating policy
Source: PNAS Nexus. 2023 Nov 11;2(12):pgad387. doi: 10.1093/pnasnexus/pgad387 (PMC10714897; doi:10.1093/pnasnexus/pgad387)
Supplement: pgad387_Supplementary_Data [file pgad387_supplementary_data.docx]

**Supplementary information**

**Trade-Offs Between Cold Protection and Air Pollution Induced Mortality of China's Heating Policy**

**Authors:**

Haofan Zhang^a,b^, Pan He^b^, Linxin Liu^f^, Hui Dai^c^, Bin Zhao^c^, Yi Zeng^d,e^, Jun Bi^a^, Miaomiao Liu^a^*, John S. Ji^f^*

**Affiliations:**

1. State Key Laboratory of Pollution Control and Resource Reuse, School of the Environment, Nanjing University, Nanjing, China
2. School of Earth and Environmental Sciences, Cardiff University, Cardiff, UK
3. Department of Building Science, School of Architecture, Tsinghua University, Beijing, China
4. Center for Healthy Aging and Development Studies, Raissun Institute for Advanced Studies, National School of Development, Peking University, Beijing, China
5. Center for the Study of Aging and Human Development and Geriatrics Division, Medical School of Duke University, Durham, NC, U.S.A
6. Vanke School of Public Health, Tsinghua University, Beijing, China

*** Correspondence to:**

**Dr. John S. Ji,** Vanke School of Public Health, Tsinghua University, Beijing, China, johnji@tsinghua.edu.cn

**Dr. Miaomiao Liu**, State Key Laboratory of Pollution Control and Resource Reuse, School of the Environment, Nanjing University, Nanjing, China, [liumm@nju.edu.cn](mailto:liumm@nju.edu.cn)

**Keywords：**

Winter heating, China, health risk assessment, air pollution, protection against low temperatures, mortality, older adult

**Table S1. Baseline characteristics of participants included in long-term exposure analysis**

|  |  | **Winter heating areas in *Huai* provinces** | **Non-winter heating areas in *Huai* provinces** | ***Huai* Provinces** |
| --- | --- | --- | --- | --- |
|  |  | **(N=1813)** | **(N=3521)** | **(N=5334)** |
| **Annual average temperature (°C)** | |  |  |  |
|  | **Mean (SD)** | 14.76 (0.88) | 16.13 (0.93) | 15.67 (1.12) |
| **Annual temperature variability (°C)** | |  |  |  |
|  | **Mean (SD)** | 9.81 (0.69) | 9.24 (0.51) | 9.43 (0.63) |
| **Summer average temperature (°C)** | |  |  |  |
|  | **Mean (SD)** | 25.66 (0.84) | 26.45 (0.87) | 26.18 (0.93) |
| **Summer temperature variability (°C)** | |  |  |  |
|  | **Mean (SD)** | 3.55 (0.46) | 3.58 (0.38) | 3.57 (0.41) |
| **W****inter average temperature (°C)** | |  |  |  |
|  | **Mean (SD)** | 2.57 (1.47) | 4.81 (1.43) | 4.05 (1.79) |
| **Winter temperature variability (°C)** | |  |  |  |
|  | **Mean (SD)** | 4.40 (0.82) | 4.07 (0.97) | 4.18 (0.93) |
| **Contemporaneous NDVI** | |  |  |  |
|  | **Mean (SD)** | 0.43 (0.23) | 0.45 (0.21) | 0.45 (0.22) |
| **1-year average PM_2.5_ (µg/m³)** | |  |  |  |
|  | **Mean (SD)** | 91.90 (16.36) | 79.22 (13.93) | 83.53 (15.98) |
| **Age** |  |  |  |  |
|  | **Mean (SD)** | 87.13 (12.06) | 84.98 (12.16) | 85.71 (12.17) |
|  |  |  |  |  |
| **Sex** |  |  |  |  |
|  | **Male** | 694 (38.3%) | 1432 (40.7%) | 2126 (39.9%) |
|  | **Female** | 1119 (61.7%) | 2089 (59.3%) | 3208 (60.1%) |
| **Ethnicity** |  |  |  |  |
|  | **Han Chinese** | 1793 (98.9%) | 3497 (99.3%) | 5290 (99.2%) |
|  | **Ethnic minority** | 20 (1.1%) | 24 (0.7%) | 44 (0.8%) |
| **Marital status** | |  |  |  |
|  | **Currently married and living with spouse** | 501 (27.6%) | 1134 (32.2%) | 1635 (30.7%) |
|  | **Not married** | 1312 (72.4%) | 2387 (67.8%) | 3699 (69.3%) |
| **School years** |  |  |  |  |
|  | **0 year** | 1363 (75.2%) | 2424 (68.8%) | 3787 (71.0%) |
|  | **1-6 years** | 323 (17.8%) | 791 (22.5%) | 1114 (20.9%) |
|  | **>6 years** | 127 (7.0%) | 306 (8.7%) | 433 (8.1%) |
| **Residence** |  |  |  |  |
|  | **City (Urban)** | 188 (10.4%) | 455 (12.9%) | 643 (12.1%) |
|  | **Town (Urban)** | 208 (11.5%) | 942 (26.8%) | 1150 (21.6%) |
|  | **County (Rural)** | 1417 (78.2%) | 2124 (60.3%) | 3541 (66.4%) |
| **Financial support** | |  |  |  |
|  | **Financial dependent** | 1461 (80.6%) | 2528 (71.8%) | 3989 (74.8%) |
|  | **Financial independent** | 352 (19.4%) | 993 (28.2%) | 1345 (25.2%) |
| **Smoking status** | |  |  |  |
|  | **Current** | 313 (17.3%) | 690 (19.6%) | 1003 (18.8%) |
|  | **Former** | 252 (13.9%) | 465 (13.2%) | 717 (13.4%) |
|  | **Never** | 1248 (68.8%) | 2366 (67.2%) | 3614 (67.8%) |
| **Drinking status** | |  |  |  |
|  | **Current** | 255 (14.1%) | 820 (23.3%) | 1075 (20.2%) |
|  | **Former** | 151 (8.3%) | 314 (8.9%) | 465 (8.7%) |
|  | **Never** | 1407 (77.6%) | 2387 (67.8%) | 3794 (71.1%) |
| **Exercise** |  |  |  |  |
|  | **Current** | 397 (21.9%) | 841 (23.9%) | 1238 (23.2%) |
|  | **Former** | 98 (5.4%) | 166 (4.7%) | 264 (4.9%) |
|  | **Never** | 1318 (72.7%) | 2514 (71.4%) | 3832 (71.8%) |

**Table S2. Baseline characteristics of participants included in short-term exposure analysis**

|  |  | **Winter heating areas in *Huai* provinces** | **Non-winter heating areas in *Huai* provinces** | ***Huai* Provinces** |
| --- | --- | --- | --- | --- |
|  |  | **(N=2070)** | **(N=4597)** | **(N=6667)** |
| **Daily max temperature (°C)** | |  |  |  |
|  | **Median [IQR]** | 17.70 [18.80] | 19.90 [16.90] | 19.30 [17.40] |
| **Season** |  |  |  |  |
|  | **Autumn** | 532 (25.7%) | 1212 (26.4%) | 1744 (26.2%) |
|  | **Spring** | 423 (20.4%) | 1027 (22.3%) | 1450 (21.7%) |
|  | **Summer** | 425 (20.5%) | 997 (21.7%) | 1422 (21.3%) |
|  | **Winter** | 690 (33.3%) | 1361 (29.6%) | 2051 (30.8%) |
| **Age group** |  |  |  |  |
|  | **<80** | 99 (4.8%) | 240 (5.2%) | 339 (5.1%) |
|  | **[80,89)** | 404 (19.5%) | 950 (20.7%) | 1354 (20.3%) |
|  | **[90,99)** | 711 (34.3%) | 1738 (37.8%) | 2449 (36.7%) |
|  | **>99** | 856 (41.4%) | 1669 (36.3%) | 2525 (37.9%) |
| **Sex** |  |  |  |  |
|  | **Male** | 721 (34.8%) | 1803 (39.2%) | 2524 (37.9%) |
|  | **Female** | 1349 (65.2%) | 2794 (60.8%) | 4143 (62.1%) |
| **Ethnicity** |  |  |  |  |
|  | **Han Chinese** | 2037 (98.4%) | 4536 (98.7%) | 6573 (98.6%) |
|  | **Ethnic minority** | 24 (1.2%) | 56 (1.2%) | 80 (1.2%) |
| **Marital status** | |  |  |  |
|  | **Currently married and living with a spouse** | 350 (16.9%) | 901 (19.6%) | 1251 (18.8%) |
|  | **Not married** | 1719 (83.0%) | 3694 (80.4%) | 5413 (81.2%) |
| **School years** | |  |  |  |
|  | **0 year** | 1671 (80.7%) | 3391 (73.8%) | 5062 (75.9%) |
|  | **1-6 years** | 301 (14.5%) | 928 (20.2%) | 1229 (18.4%) |
|  | **>6 years** | 85 (4.1%) | 258 (5.6%) | 343 (5.1%) |
| **Residence** |  |  |  |  |
|  | **City** | 192 (9.3%) | 713 (15.5%) | 905 (13.6%) |
|  | **Town** | 375 (18.1%) | 1576 (34.3%) | 1951 (29.3%) |
|  | **County** | 1503 (72.6%) | 2308 (50.2%) | 3811 (57.2%) |
| **Financial support** | |  |  |  |
|  | **Financial dependent** | 1554 (75.1%) | 3012 (65.5%) | 4566 (68.5%) |
|  | **Financial independent** | 170 (8.2%) | 589 (12.8%) | 759 (11.4%) |
| **Smoking status** | |  |  |  |
|  | **Current** | 290 (14.0%) | 805 (17.5%) | 1095 (16.4%) |
|  | **Former** | 292 (14.1%) | 667 (14.5%) | 959 (14.4%) |
|  | **Never** | 1486 (71.8%) | 3121 (67.9%) | 4607 (69.1%) |
| **Drinking status** | |  |  |  |
|  | **Current** | 266 (12.9%) | 1103 (24.0%) | 1369 (20.5%) |
|  | **Former** | 170 (8.2%) | 464 (10.1%) | 634 (9.5%) |
|  | **Never** | 1631 (78.8%) | 3026 (65.8%) | 4657 (69.9%) |
| **Exercise** |  |  |  |  |
|  | **Current** | 425 (20.5%) | 931 (20.3%) | 1356 (20.3%) |
|  | **Former** | 136 (6.6%) | 256 (5.6%) | 392 (5.9%) |
|  | **Never** | 1505 (72.7%) | 3399 (73.9%) | 4904 (73.6%) |

**Table S3. Hazard ratios and 95% confidence intervals for seasonal temperature and mortality under different heating status**

|  | **Winter heating areas in *Huai* provinces** | | **Non-winter heating areas in *Huai* provinces** | |
| --- | --- | --- | --- | --- |
|  | **(N=1813)** | | **(N=3521)** | |
| **PM_2.5_ (10 µg/m³)** | 1.129*** | 1.121*** | 1.133*** | 1.116*** |
|  | (0.018) | (0.018) | (0.017) | (0.176) |
| **Annual average temperature (1°C)** | 0.983 |  | 1.111*** |  |
|  | (0.030) |  | (0.023) |  |
| **Annual temperature variability (1°C)** | 1.273*** |  | 1.423*** |  |
|  | (0.050) |  | (0.044) |  |
| **Summer average temperature (1°C)** |  | 1.168*** |  | 1.292*** |
|  |  | (0.033) |  | (0.026) |
| **Summer temperature variability (1°C)** |  | 1.111*** |  | 1.174*** |
|  |  | (0.052) |  | (0.044) |
| **Winter average temperature(1°C)** |  | 0.897*** |  | 0.862*** |
|  |  | (0.017) |  | (0.016) |
| **Winter temperature variability(1°C)** |  | 1.076*** |  | 1.212*** |
|  |  | (0.033) |  | (0.024) |

**Table S4. Hazard ratios and 95% confidence intervals for winter heating and mortality adjusted different air pollution exposure assessment**

| **Air pollution exposure assessment** | **Winter heating** | | | **PM_2.5_** | | |
| --- | --- | --- | --- | --- | --- | --- |
|  | HR | 95% CI | p value | HR | 95% CI | p value |
| **City-level indoor PM_2.5_** | 0.780 | (0.722, 0.843) | *** | 1.004 | (1.000, 1.009) | * |
| **Average of indoor and outdoor PM_2.5_** | 0.769 | (0.712, 0.830) | *** | 1.020 | (1.013, 1.026) | *** |
| **Weighted average of indoor and PM_2.5_** | 0.792 | (0.733, 0.856) | *** | 1.014 | (1.005, 1.023) | *** |

All models adjusted age, gender, ethnicity, urban/rural residence, education, marital status, financial support, smoking status, alcohol consumption, physical activity and air pollution.

Average of indoor and outdoor PM_2.5_ is the mean of indoor and outdoor PM_2.5_ concentrations.

Weighted average of indoor and PM_2.5_ is equal to 87% of indoor PM_2.5_ concentrations plus 13% outdoor PM_2.5_ concentrations. (People spend 87% of their time indoors)

**Figure S1. Risk estimates for the overall temperature-mortality relationship stratified by heating status in *Huai* Provinces**


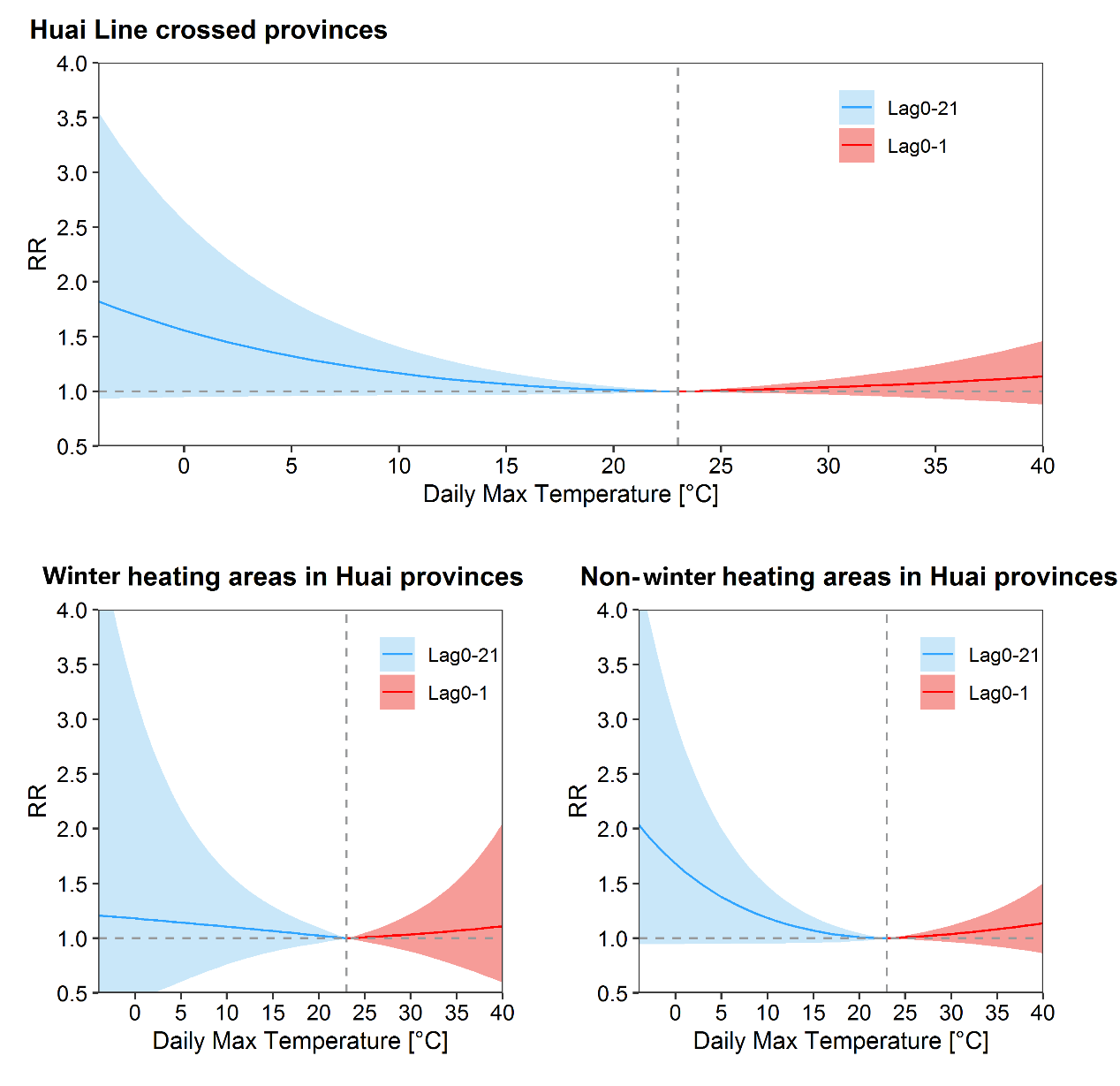


**Figure S2. Survival Probabilities over time**


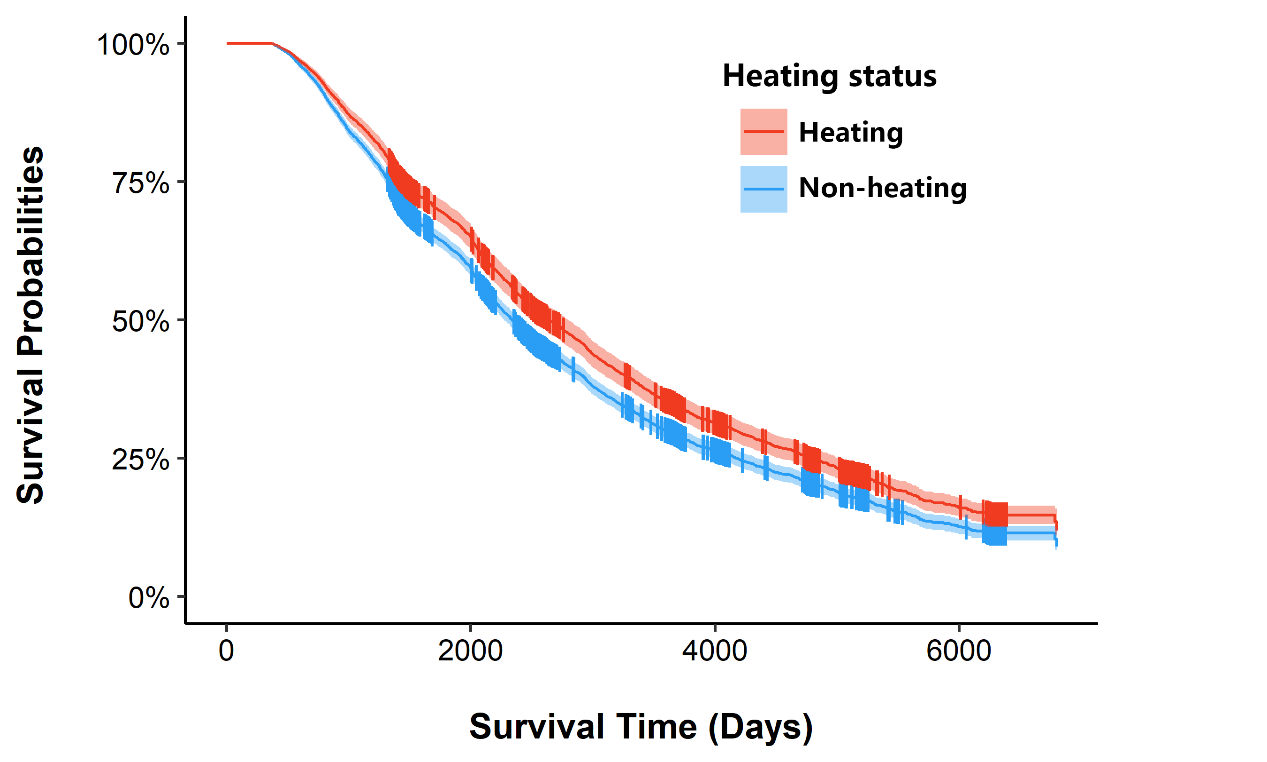


**Figure S3. Hazard ratios and 95% confidence intervals for winter heating and mortality in different populations**


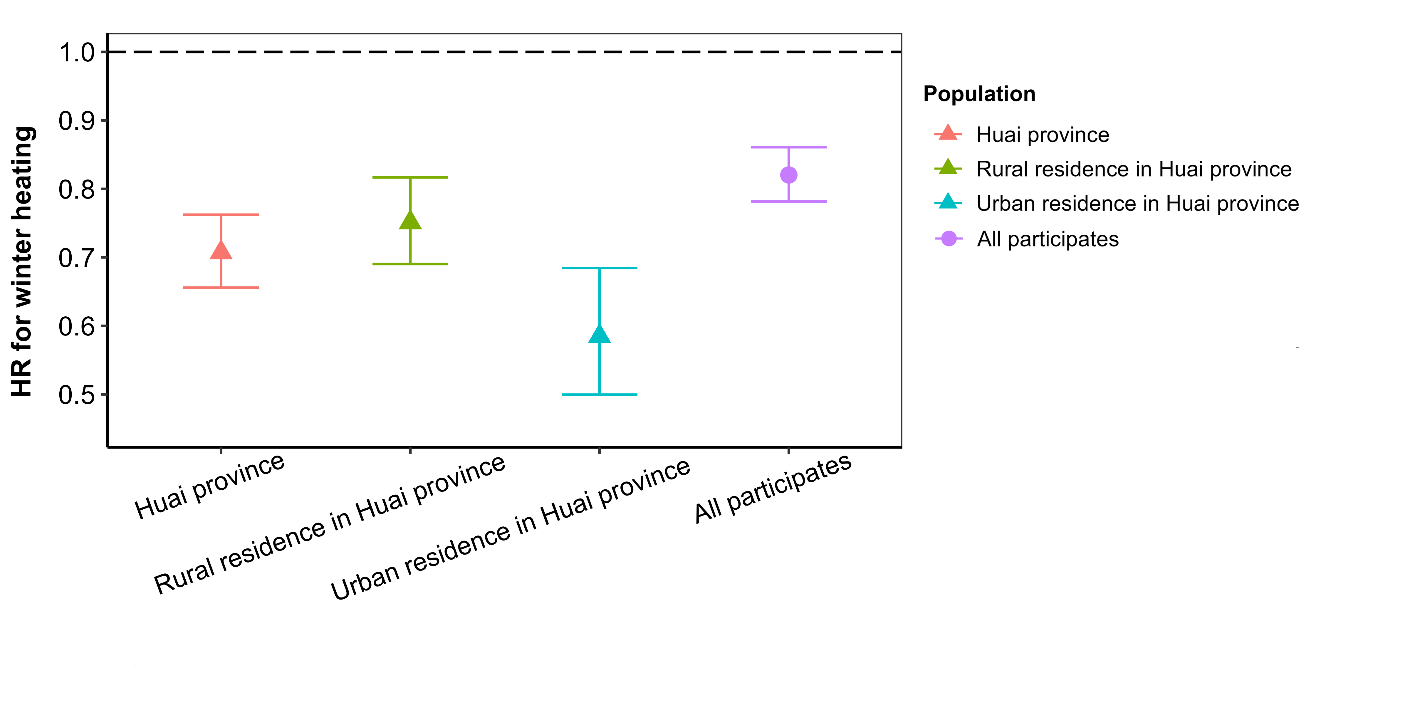


All models adjusted age, gender, ethnicity, urban/rural residence, education, marital status, financial support, smoking status, alcohol consumption, physical activity, residential greenness_,_ residential PM_2.5,_ summer average temperature, summer temperature variability, winter average temperature and winter temperature variability.
